# Simulating Quantitative Cellular Responses Using Asynchronous Threshold Boolean Network Ensembles

**DOI:** 10.1186/1752-0509-5-109

**Published:** 2011-07-11

**Authors:** John Jack, John F Wambaugh, Imran Shah

**Affiliations:** 1 National Center for Computational Toxicology, Office of Research and Development, U.S. Environmental Protection Agency, Research Triangle Park, North Carolina, USA

## Abstract

**Background:**

With increasing knowledge about the potential mechanisms underlying cellular functions, it is becoming feasible to predict the response of biological systems to genetic and environmental perturbations. Due to the lack of homogeneity in living tissues it is difficult to estimate the physiological effect of chemicals, including potential toxicity. Here we investigate a biologically motivated model for estimating tissue level responses by aggregating the behavior of a cell population. We assume that the molecular state of individual cells is independently governed by discrete non-deterministic signaling mechanisms. This results in noisy but highly reproducible aggregate level responses that are consistent with experimental data.

**Results:**

We developed an asynchronous threshold Boolean network simulation algorithm to model signal transduction in a single cell, and then used an ensemble of these models to estimate the aggregate response across a cell population. Using published data, we derived a putative crosstalk network involving growth factors and cytokines - i.e., Epidermal Growth Factor, Insulin, Insulin like Growth Factor Type 1, and Tumor Necrosis Factor α - to describe early signaling events in cell proliferation signal transduction. Reproducibility of the modeling technique across ensembles of Boolean networks representing cell populations is investigated. Furthermore, we compare our simulation results to experimental observations of hepatocytes reported in the literature.

**Conclusion:**

A systematic analysis of the results following differential stimulation of this model by growth factors and cytokines suggests that: (a) using Boolean network ensembles with asynchronous updating provides biologically plausible noisy individual cellular responses with reproducible mean behavior for large cell populations, and (b) with sufficient data our model can estimate the response to different concentrations of extracellular ligands. Our results suggest that this approach is both quantitative, allowing statistical verification and calibration, and extensible, allowing modification and revision as guided by experimental evidence. The simulation methodology is part of the US EPA Virtual Liver, which is investigating the effects of everyday contaminants on living tissues. Future models will incorporate additional crosstalk surrounding proliferation as well as the putative effects of xenobiotics on these signaling cascades within hepatocytes.

## Background

### Motivation

Thousands of chemicals are used in commerce and evaluating their public health risk remains a challenging problem [1,2]. Much of our knowledge about mechanisms of toxicity is based on evidence from *in vivo *animal studies and *in vitro *experiments, where we can begin to unravel some of the molecular signaling and transcriptional changes induced via chemical perturbation; however, there are three main issues in translating these findings to humans. First, it is often impractical to design experiments with sufficient power to detect the subtle effects of very low environmentally relevant exposures [3]. Hence most observations about chemical effects are at relatively high concentrations that cannot be easily used to quantify the risk of long-term and low-dose exposure to complex mixtures of chemicals [4]. Second, since the molecular response to chemicals is not always conserved between species [5], the effects observed in rodents cannot be directly extrapolated from rodents to humans without additional mechanistic insight [6]. Third, toxicity is a tissue level phenomenon arising from the behaviors of heterogeneous cell populations. Understanding the complex signaling processes between these different cell types is crucial in determining toxicity potential. We are building a cell-based tissue model to estimate the quantitative population-level effects of chemical exposures [7,8]. Here we describe an asynchronous threshold Boolean network (BN) approach to model signal transduction in individual cells and to estimate tissue level responses using an ensemble of BNs.

### Boolean Networks

A BN describes a signaling network as a digital circuit in which logical elements (proteins or genes) are either 'ON' or 'OFF'. The temporal evolution of the signaling network is calculated using a set of Boolean functions (AND, OR, NOT) to model regulatory interactions. Since they offer a biologically relevant and computationally efficient formalism for analyzing the relationship between molecular network topology and function, BNs have been used extensively to simulate the behavior of cells based on their network activity. Genetic regulatory networks have been particularly amenable to this formalism due to the binary nature of gene activation [9]. The availability of large-scale transcriptional profiles spurred more recent applications of deterministic [10] and probabilistic [11] BNs for reconstructing and simulating genetic regulatory networks. Additionally, BNs have been used for modeling the cell cycle [12-15]; cell proliferation [16-18]; apoptosis [19]; and cancer [20]. BNs can be used to represent the binary activity of molecular species across cell populations (*in vitro *and *in vivo*). One of the limitations of BNs is that they cannot readily estimate continuous functional responses, i.e., quantitative dose-response, which are fundamentally important in pharmacology and toxicology.

### Cancer Pathways

Liver cancer is a frequent outcome in testing the long-term effects of chemicals in rodents [21] and often considered in regulatory decisions [22]. Since the mechanisms of carcinogenesis are poorly understood, it is difficult to translate chemical effects from rodents to humans. Cancer is believed to arise due to a breakdown of the homeostatic processes that maintain balance between cell death and division [23]. Some chemicals (called mutagens) can alter cell phenotypes by damaging DNA resulting in harmful mutations that can spur the activation of oncogenes. Nongenotoxic carcinogens, on the other hand, can act via insidious mechanisms that suppress apoptosis or to stimulate cell proliferation.

It has been suggested that nongenotoxic carcinogens may increase hepatocyte proliferation by perturbing the crosstalk network regulated by growth factors and cytokines [24]. Crosstalk refers to the sequence of protein regulation activated by any one growth factor or cytokine ligand overlapping with the sequences of other ligands, which allows for complex behavior. The presence of crosstalk allows a cell to behave as a multiplexer, integrating multiple signals to select from many possible outcomes, such as cell cycle initiation and progression.

A number of computational models have been proposed for simulating cell proliferation [12-18,25], however, BNs have not been extensively used in modeling chemical induced toxicity or in hepatic biology. In order for a chemical to produce a chronic or acute tissue level effect, there must be some level of perturbed protein activity in the signal transduction of one or more cells. We are evaluating BNs for modeling early molecular signaling events in hepatocytes that lead to proliferative changes, which are key events in carcinogenesis. Hence, our initial objective is to model some of the normal cues, i.e. homeostatic processes, that can stimulate healthy, quiescent hepatocytes (G0) into entering the cell cycle (G1).

Technological advancements such as flow cytometry and high content screening have made it possible to measure protein levels with single cell resolution. Experimental observations suggest that protein levels within cells may exhibit a switch-like 'all' or 'nothing' ('ON' or 'OFF') response - for example, p53 response to DNA damage [26,27], TNFα stimulation [28,29], MAPK signaling events [30], and drug treatment [31]. These types of observations serve as a foundation for the hypothesis that a Boolean representation is sufficient for describing the molecular multiplicities of individual cells in a simulation framework. Next, we assume that the aggregate activity of molecules across a population of hundreds, thousands or millions of cells can be used to estimate tissue level responses.

### Key Contributions

Our work is based on two extensions of asynchronous BNs, which employ a non-deterministic updating scheme. First, we use threshold functions to calculate the activation of each protein in our model. This technique has been applied to other systems [32,33], and it provides a simple representation and adjustable parameter for investigating the interactions between signaling molecules. Second, we model an ensemble of BNs to simulate the quantitative responses of thousands of cells. As such, we can estimate dose dependent responses of cell populations. We defined the topology of the BN semi-automatically using structured information about canonical signaling network from a public pathway repository. Here we describe our initial results on the reproducibility of asynchronous threshold Boolean network ensembles and their potential utility for estimating quantitative time- and concentration-dependent biological responses.

## Results

### Cell Signaling Network Construction

We used the Science Signaling database (or STKE) [34-37] to construct the protein signaling network. The canonical pathways in the network include: Epidermal Growth Factor (EGF) signaling, Insulin (INS) signaling, Insulin like Growth Factor type 1 (IGF-1) signaling, and Tumor Necrosis Factor alpha (TNFα) signaling. The number of proteins and molecular interactions in each of these pathways are summarized in Table 1. We performed several steps to systematically build a crosstalk network from these canonical pathways. First, we combined all of the proteins and interactions from the four pathways into one integrated molecular interaction network. After filtering for uniqueness among proteins and interactions, we produced a non-redundant crosstalk network with 102 proteins and 150 interactions. Second, we excluded the proteins and interactions that did not lead to c-Jun and c-Fos activity, which are important components in the formation of the activator protein 1 (AP-1) transcription factor complex.

**Table 1:** Pathway Information for Building Crosstalk Model

***Pathway***	***Proteins***	***Interactions***
**Epidermal Growth Factor (EGF)**	49	66
**Insulin-like Growth Factor Type 1 (IGF-1)**	8	7
**Tumor Necrosis Factor alpha (TNFα)**	24	37
**Insulin (INS)**	50	65
**Merge Four Pathways**	102	150
**Subgraph: all paths from receptors to c-Jun or c-Fos**	55	89
**Final network: remove with in degree = 0**	46	77

We combined four pathways from the Science Signaling Database (STKE). The first four rows of the table show the numbers of proteins and interactions in each pathway. The last three rows show the numbers of proteins and interactions as we merge and simplify the network.

**Figure 1:**
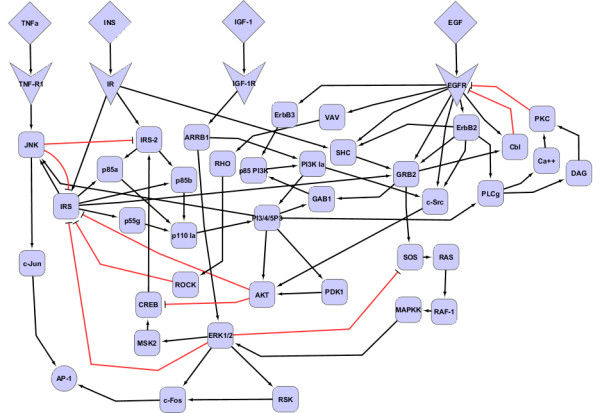
**Putative Crosstalk Network for Simulation**. The network shows signaling interactions due to extracellular ligands including: EGF, IGF-1, INS, and TNFα. The pathways share common adapter proteins (e.g., SHC and GRB2) as well as downstream signals (ERK1/2 and c-Fos/c-Jun activation).

In our initial model, we focused on early signaling events in cell proliferation and did not consider gene expression changes which lead to mitosis. Hence, we assumed that AP-1 formation, encoded as a c-Jun/c-Fos dimer, is an early marker of cell cycle progression. This allowed us to further simplify the network by removing all proteins and interactions that are not on a pathway from one of the four receptors to either c-Jun or c-Fos. Furthermore, we manually removed an additional six proteins with in degree less than 2. We did, however, leave some proteins with in degree less than two: the extracellular ligands and their receptors, as well as Rat Sarcoma (RAS), ribosomal s6 kinase (RSK), v-erb-b2 erythroblastic leukemia viral oncogene homolog 2 (ErbB2) and homolog 3 (ErbB3), Rho GTPase (RHO), p55 gamma (p55g), Vav proto-oncogene (VAV), c-Jun, mitogen- and stress-activated protein kinase 2 (MSK2), mitogen activated protein kinase kinase (MAPKK), phosphoinositide-dependent kinase 1 (PDK1), and 1,2-Diacylglycerol (DAG). These molecules are implicated in the EGF signaling pathway, which was simulated and compared to *in vitro *data, except p55g which is involved INS pathway. Finally, we included in the model a molecular species representing AP-1 transcription factor complex formation, by adding two additional interactions involving the c-Jun and c-Fos dimerization. The final biochemical interaction network contained 46 proteins and 77 interactions. The protein signaling network in Figure 1 was drawn with Cytoscape [38], an open source tool convenient for visualizing large scale networks.

### Simulating individual cellular responses

We used the biochemical interaction network in Figure 1 to describe the response of an individual hepatocyte to the growth factors (EGF, IGF-1 and INS) and the inflammatory cytokine (TNFα). In order to simulate the dynamics of signal transduction, we translated the biochemical interaction network into a threshold BN. As in a traditional BN approach, we assumed that: (a) proteins in the network are described by one of two states, active (ON) or inactive (OFF) and, (b) interactions result in either the activation or inhibition of output proteins by input proteins. Our approach deviates from traditional BNs in three important ways. First, we replace the logical operators with an integer summation function that incorporates an activation threshold. This allows us to adjust the activating potential of each protein in the network. Second, we employ a nondeterministic, asynchronous updating scheme (see Methods), which can simulate biological 'noise' observed in protein signaling cascades. Third, we provide a method for using Hill functions for calibrating the probability of activation for proteins in the network, which can be calibrated with concentration-response data.

**Figure 2:**
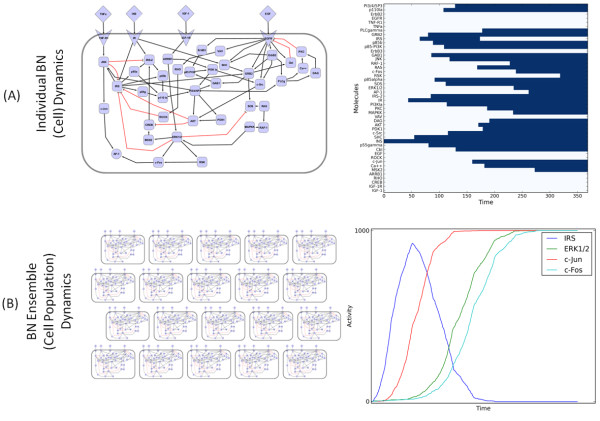
**Overview of Boolean Network Ensembles**. (A) A single synthetic cell as a Boolean network (left) along with discrete dynamic response of proteins in one simulated cell to INS treatment (right). (B) The aggregate activity profiles of four proteins (right) across 1000 simulated cells (a replicate) in response to INS treatment. The abscissa shows time steps and the simulation terminates when all cells have reached steady state.

In our methodology, the signaling network in a single cell is represented as one asynchronous threshold BN. Figure 2(A) illustrates the model of an individual cell as a BN and its discrete dynamic response following treatment with INS. The BN is constructed from the model shown in Figure 1 (see Methods). The temporal evolution of protein activity in one BN is visualized as a heatmap in Figure 2(A) (right panel). The abscissa of the heatmap shows the simulation time steps (denoted as τ). At τ = 0 the cell was 'treated' with INS by switching the ligand from OFF to ON. Each column in the heatmap shows the dynamic changes in the state of proteins (given in the ordinate) at time steps following INS treatment. The simulation continues until (τ = 369) when it reaches a steady state, which involves the activation of the AP-1 transcription factor complex. The discrete profile of each protein shows the asynchronous dynamics of signal transduction through the insulin receptor (IR) in our crosstalk model (shown in Figure 1). In other words, Figure 2a depicts a putative sequence of signaling events that could occur in a single hepatocyte after insulin stimulation. This result is qualitatively concordant with *in vitro *observations on AP-1 formation following insulin treatment [39]. Since the output of a single BN is binary, however, it is difficult to evaluate the activation of AP-1 to different concentrations of INS or other ligands for a single cell.

### Simulating cell population responses

In order to estimate the quantitative response to treatments, we assume that cell populations can be modeled as an ensemble of asynchronous BNs. This allows us to estimate the dynamic response across a simulated biological sample as the aggregate activity of each protein across thousands of BNs (see Methods). Hence, an ensemble of BNs can be considered a simulated 'replicate' as illustrated in Figure 2(B). We investigated the response of an ensemble of 1000 asynchronous BNs to treatment with INS (including the BN depicted in Figure 2(A) until all BNs reached a steady state. The resulting aggregate activity profiles of IRS, c-Jun, c-Fos, and ERK1/2 are shown in Figure 2b (right panel). These trends captured by the simulated BN ensembles appear similar to experimental data from molecular assays performed on *in vivo *and *in vitro *replicates (which contain a large number of cells). While this requires additional quantitative and mechanistic evaluation, it is important to note that such continuous protein activity profiles cannot be generated using traditional BNs. Before further evaluation with experimental data we analyzed the reproducibility of our approach with respect to the network depicted in Figure 1.

### Reproducibility of Protein Activity Profiles

We systematically evaluated the reproducibility of the asynchronous BN ensembles of the model shown in Figure 1 by analyzing their response to different treatment conditions. For each treatment condition, we simulated 100 replicates with 1000 cells per replicate (i.e., 100,000 cells per treatment condition). Each treatment condition is defined by combinatorial stimulation of the four extracellular ligand cues: (i) EGF, TNFα, IGF-1, and INS combined; (ii) TNFα and INS; (iii) TNFα and IGF-1; (iv) TNFα and EGF; (v) EGF only; (vi) IGF-1 only; (vii) INS only; (viii) TNFα only; (ix) and no active extracellular ligands (the control group). We assumed that each cell is exposed to enough ligand in order to activate a sufficient number of receptors for signal propagation. Hence, for each of the simulated treatment conditions 100% of the cells receive stimulation. Moreover, following the logic of Boolean abstraction of protein concentration, we assumed that the ligand is switched 'ON' in every cell upon initialization.

**Figure 3:**
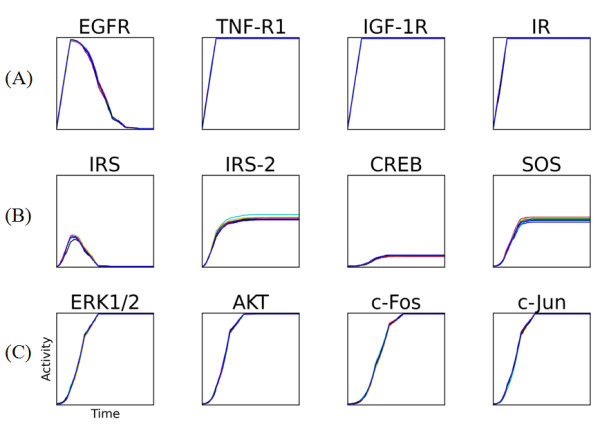
**Simulated Replicate Protein Activity Profiles**. The protein activity profiles after treatment with all ligands for 1000 simulated cells. The graphs show the time course response of a subset of proteins in the model including: (A) receptor tyrosine kinases, (B) select intermediate signaling proteins, and (C) select transcription factor proteins including c-Fos and c-Jun. For instance, EGFR shows an initial increase in activity followed by inactivation due to feedback inhibition, which is consistent with our knowledge of receptor internalization and ubiquitination. We have not included similar feedback for the other receptor in the current model. Similarly, IRS is a hub signaling protein with a number of potential inhibitors and some of these are represented in our model. The activation of the MAPKs (ERK1/2) and transcription factors (AKT, c-Fos, c-Jun) in our simulation highlights the putative signaling cascade responsible for activating immediate early genes, which is a key step in cell cycle progression into S phase.

**Figure 4:**
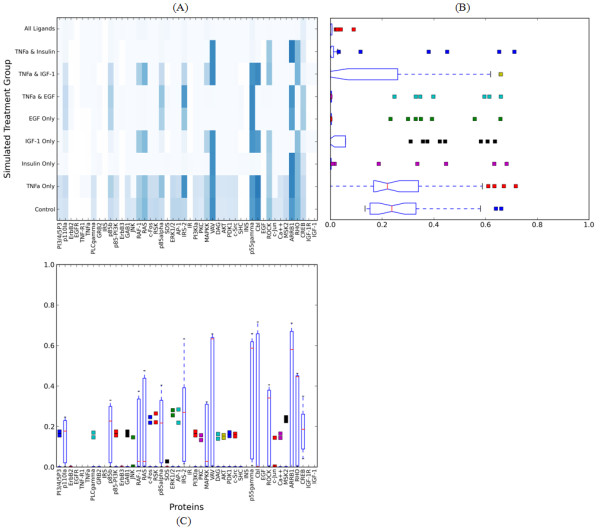
**Reproducibility of Protein Activities Across Simulated Replicates**. (A) The heatmap shows the coefficient of variation for protein activity (columns) following different treatments (rows). The color intensity increases with decreasing reproducibility. The nine treatments shown in the rows are (i) EGF, TNFα, IGF-1, and INS combined; (ii) TNFα and INS; (iii) TNFα and IGF-1; (iv) TNFα and EGF; (v) EGF only; (vi) IGF-1 only; (vii) INS only; (viii) TNFα only; (ix) and no active extracellular ligands (Control). Each cell in the heatmap represents the coefficient of variation for the activity of one protein across 100 replicates with 1000 cells/replicate (a total of 100,000 Boolean network simulations). (B) The distribution of CV across each treatment condition. (C) The distribution of CV across each protein.

Figure 3(A) shows the dynamic responses of the simulated replicates in the treatment group (i), the combined stimulation of all extracellular ligands. Each of the 12 plots shows the activity profile for one protein from a random sampling of eight replicates. Even though the activity profile of each replicate is noisy, the overall trend across the eight replicates in the (i) treatment group appears to be reproducible. To analyze this further, we calculated the coefficient of variation (CV) for every treatment group (see Methods). These results are summarized in the heatmap in Figure 4(A). The rows of the heatmap correspond to the treatment groups and the columns to proteins in our model. Each cell shows the normalized CV across all proteins and treatment where increasing color saturation signifies decreasing reproducibility. For instance, the simulated treatment with all ligands produces highly reproducible changes in steady state protein activities, whereas there is considerable variation in the absence of any treatment. Overall, the protein activation across replicates is generally reproducible and well within the range of actual experiments [40].

In Figure 4(B), we show the distribution of the CV for the steady state protein activities across all treatment groups as a box and whisker plot. Similarly, Figure 4(C) shows the reproducibility across the proteins for each treatment group. Whereas the heatmap of Figure 4(A) shows information on the CV per protein per treatment condition, the plots in Figures 4(B) and 4(C) visualize the overall behavior of the model across each treatment condition and protein, respectively. We found that treatment conditions (iii), (vi), (viii) and (ix) - IGF-1 and TNFα, IGF-1 only, TNFα only, and the untreated control group - were the least reproducible in comparison to all other treatment conditions. For the control group, a possible explanation for the reproducibility result is that the median activity of proteins in the control group is very low. As a result, signaling molecules other than ligands and receptors have a very low probability of being active at initialization, in order to simulate a background level of hepatocyte proliferation. This very low mean value has the effect of inflating the CV. In the case of IGF-1 or TNFα, the low reproducibility could be due to the inclusion of fewer reactions than the two other growth factors (EGF and INS). Hence, stimulating with IGF-1 or TNFα may not sufficiently stimulate the individual BNs for the entire ensemble to synchronize in response to treatment. Similar logic governs the model reproducibility following combined stimulation with IGF-1 and TNFα. These results also help to illuminate the sensitivity of our simulation approach to the topology of the signaling network. Importantly, the key endpoint of the model, AP-1 formation, is very reproducible across all treatment conditions.

### Comparison with Experimental Data

We used experimental data on primary hepatocytes in culture [41] for a preliminary evaluation of our simulation approach and putative crosstalk model. In this experiment, rat primary hepatocytes were treated with varying concentrations of EGF and/or TNFα, and then the proportion of cells entering S phase (DNA Synthesis) was measured using Bromodeoxyuridine (5'-bromo-2'-deoxyuridine, BrdU). Although we do not explicitly model S phase in our network, the formation of the AP-1 transcription factor complex is believed to precede S phase in cell cycle progression. Hence, we assumed the formation of AP-1 to be a potential surrogate of S phase and, therefore, correlated with BrdU incorporation. We adjusted the probabilities of activation for proteins in our network in order to closely approximate the levels of BrdU incorporation in the absence of any treatment (control group). Further details on the calibration of the model are described in the Methods section.

We simulated the effects of different treatments on AP-1 formation.

**Figure 5:**
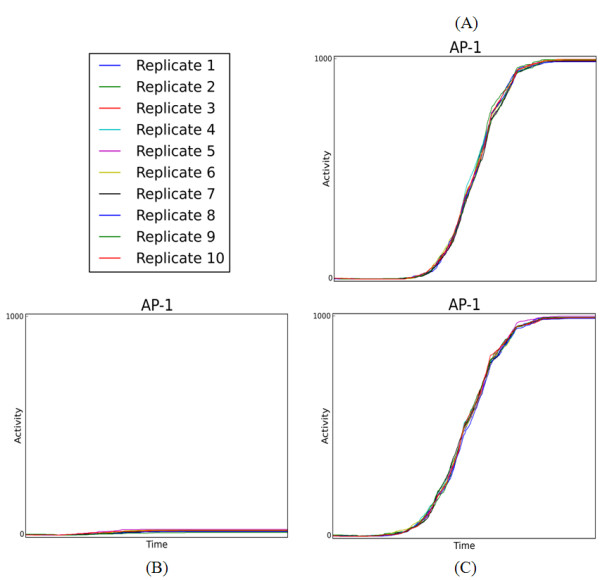
**AP-1 Activity Response Profiles Across Simulated Replicates to Different Treatment Conditions**. Each replicate line represents the activity of AP-1 across 1000 simulated cells. There are three different treatment conditions: (A) EGF only, (B) TNFα only or (C) EGF and TNFα.

**Figure 6:**
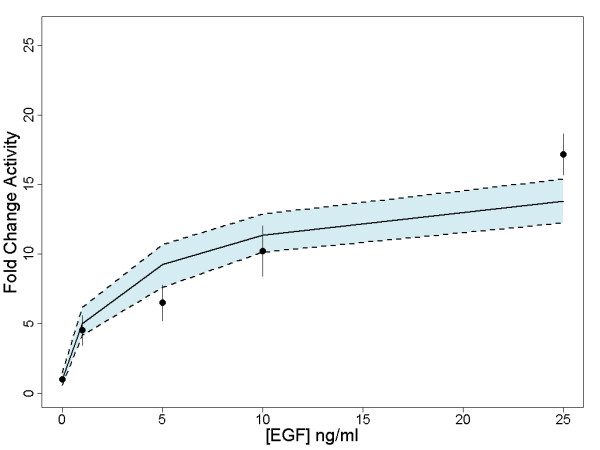
**Quantitative Comparison Between Simulation and Experimental Results**. The quantitative comparison of simulated concentration response data with experimental data on EGF. The solid line represents the mean steady state fold change of AP-1 activity (relative to control) across 100 replicates with 1000 cells per replicate, simulated over a range of values from 0 to 25 ng/ml EGF. The 95% confidence interval across replicates is in blue. The experimental results of BrdU incorporation are plotted with standard deviation as points.

Figure 5 shows the results of simulating 10 replicates for each of the treatment conditions including: combined EGF and TNFα, EGF only and TNFα only. The graphs in Figure 5 show the predicted activity profile of the AP-1 transcription factor complex across simulation time steps with the probability of activation for the treatment molecules set to 100%. Next, we simulated the concentration dependent effects of EGF stimulation. The steady state levels of AP-1 activity are shown in Figure 6 with the experimental data on BrdU incorporation for different treatment conditions. For each treatment condition, we simulated 100 replicates with 1000 cells per replicate. The plot in Figure 6 has a solid black line representing the mean of the fold change of AP-1 activity relative to mean of the control activity (at steady state) across all replicates. Based on the replicate data, we also report the 95% confidence interval for each plot -- the shaded blue region. The experimental data from [41] on BrdU incorporation is shown as points with the standard deviation. The simulation is able to reproduce the experimentally observed trends in DNA synthesis. As EGF is known to activate the AP-1 transcription factor complex (as a c-Jun/c-Fos dimer) in hepatocytes and other cell types [42], this result is consistent with the literature. Finally, the model did not predict the synergistic effect of EGF and TNFα stimulation on S phase. We believe this suggests mechanistic limitations in our crosstalk model that could be improved by incorporating additional mechanistic information about the downstream interactions between TNFα and EGF pathways.

## Discussion

BNs have been used extensively to model the dynamics of molecular signaling and genetic regulatory networks. Because they require the discretization of molecular activity levels, however, a BN cannot be readily used to model the continuous concentration- and time-dependent effects of treatments. To address this issue we extended traditional BNs in three ways. First, we model the molecular response of an individual cell using a BN. Hence, we assumed that the average activity of signaling molecules in individual cells exhibits a switch-like (ON or OFF) response. Although this may not always be the case, we believe it is more biologically plausible than a binary representation of tissue level molecular activities. Second, we assumed that cellular response to stimulation is nondeterministic. It has been suggested synchronous updating schemes for simulating BNs can produce spurious attractors that disappear in the presence of noise [43,44]. An asynchronous updating scheme, on the other hand, allows for variability (or noise) in cellular responses: two identical cells with the same initial configurations respond differentially to the same stimulus over time. Although asynchronous updating is not new in BNs [44], we define a probability of activation for each molecular species that is amenable to calibration and evaluation using experimental data. Third, we simulate an ensemble of asynchronous BNs to estimate the aggregate activity of each molecular species across cell populations. Our results show that this approach produces continuous responses similar to experimental observations from tissues. We believe this opens up new possibilities for estimating quantitative dose- and time-dependent responses in toxicology and in disease progression using knowledge of molecular mechanisms.

For this work, we used this simulation methodology to analyze the dynamics of a specific biochemical interaction network, which was constructed to investigate early molecular events surrounding hepatocellular proliferation. This is important because sustained cell proliferation is one of the key events in the progression of liver cancer. We find that (a) our extension of BNs yields highly reproducible results that have variability consonant with biological data and (b) our pathway-driven preliminary cytokine and growth factor protein signaling network is concordant with experimental observations on DNA synthesis in hepatocytes.

We investigated the effects of protein deletion from the network. In Additional file 1, we show a heatmap of these results. From this analysis, we believe our network is robust to the deletion of single proteins. Additionally, this information helps illustrate important signaling nodes in the network. For example, ERK1/2 and JNK appear to play important roles, since the removal of these changes the steady state values of other signaling molecules. To our surprise, the removal of IRS activity did not have a large effect on the signaling processes relative to other experimental conditions, even though the signaling molecule has a high connectivity in the graph.

A number of formalisms have been used to model the dynamics of eukaryotic cell cycle initiation/progression. Tyson and coauthors used ordinary differential equations (ODEs) to describe key cell division in frog oocytes [45]. Zielinski and colleagues used fuzzy Boolean logic to simulate receptor mediated crosstalk preceding cell proliferation in SKOV3 human epithelial cell line [46]. Similar fuzzy models have been proposed by others [47]. One advantage common to both techniques - i.e., ODEs and fuzzy logic - is that they can represent continuous or multivalued treatment concentrations. While these methods are powerful, they predict the population behavior of molecular species without emphasizing individual cellular protein activity. Our objective is to model the heterogeneous response of cell populations in order to estimate histological effects due to different treatments, which necessitates a modeling paradigm with individual cell clarity.

In order to help unravel the mechanisms of toxicity, we are compelled to investigate a simulation framework with a strong emphasis on network topology and reduced parameter space. ODE-based methods involve rate constants which are often difficult to quantify. Testing perturbations to systems of ODEs is not always straightforward with limited data. With BN dynamics, we lose some resolution of time but significantly reduce the number of parameters. We believe our technique is amenable to high throughput modeling of perturbations over a diverse chemical landscape, where the calibration of parameters can be limited due to scarce data on large numbers of environmental chemicals. We hope the modeling framework proposed will be useful in testing chemical perturbations from HTS data to generalized networks on signaling events assessed at the tissue level.

A comparable modeling framework was proposed [18] which also considered populations of networks. There are several differences in the modeling approaches. First, we use a binary representation of protein activity in a single cell while [18] used a ternary description to capture the level of protein expression relative to a population average under normal conditions. An ON/OFF representation of protein activity may not always be sufficient (e.g., caspase 8 activity) but it is generally consistent with single cell level observations (e.g. flow cytometry or high-content screening). The ternary representation used by [18] is based on western blot data on individual proteins with a comparison between treatment and control groups. While a mathematical transformation could relate one approach to another, the two techniques use a different abstraction for describing single cell biology.

Second, there is a subtle distinction between the abstraction of population level behavior between the two approaches. The authors of [18] calculated the average behavior of proteins across a set of BN whereas we use summation. This allows us to compare dose-dependent differences in potency and efficacy between treatments. Furthermore, it also enables the quantitative evaluation of population level "up-regulation" or "down-regulation" between treatment and control groups without using a ternary representation.

Third, the authors of [18] evaluate the effects of knockouts by maintaining some of the proteins in at 'control' or 'below control'. On the other hand, we consider the effects of dose-dependent perturbations in protein activities (e.g. extracellular ligands or intracellular signaling molecules) by using Hill functions to define probability of activation for certain protein(s) across the cell population. To our knowledge, no one has used this approach to incorporate concentration-response data in a BN modeling framework. The ability to reproduce and predict concentration-response data is essential for toxicological applications, bridging data from toxicity studies with systems biology to anticipate adverse outcomes.

We did not consider edge weights as an adjustable parameter for the model. All edges are weighted equally (set to 1.0). Modifying the edge weights would change the dynamics of the simulation. For example, in [48], the authors use a sigmoid function of the weighted sum to determine the probability per node in the propagation of the signal. Their technique offers a unique method for additional stochasticity to a threshold modeling framework. Modifying edge weights would require careful consideration of the updating scheme (describe in Methods), which would be affected if the edge weights were allowed to vary throughout simulation.

**Figure 7:**
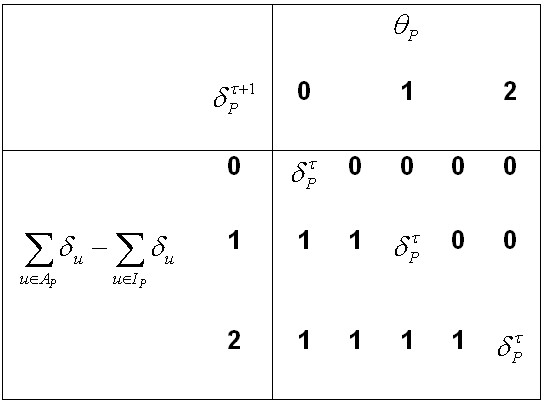
**Truth Table for Update Scheme**. The truth table illustrates how we calculate the activity of a protein (*P*) after one time step  based on the aggregate input (column 1) and differential threshold values (columns 2 - 7). For example, row 2 shows that the activity of *P *remains the same as the previous time step () when the aggregate input is 0 and *θ_P _*= 0, and  for all other threshold values. Similarly, the other row show of the consequence of varying the threshold for other values for the aggregate input. Note the distinction between integer and non-integer choices for the threshold.

The thresholds described in the methods section provide a tunable parameter for investigating the signaling interactions. Each protein has a threshold value which defines the biochemical interaction surrounding its activity - that is, the logic underlying the interaction of the activating and inhibiting molecules. To illustrate the effects of modifying the threshold of a molecule in a network, we provide Figure 7: a truth table for variations on threshold values. The truth table displays the differences in the activity of a molecule, *P*, after one update (time step), as a function of the input value - the sum of the states of the inhibiting proteins subtracted from the sum of the states of the activating proteins - and the threshold value. Setting the threshold to an integer value allows for the molecule to maintain its current state whereas, following the discussion of edge weights, setting the threshold to a noninteger value will force a decisions for a (new) value of 0 or 1.

For the crosstalk network model investigated in this paper, randomly increasing the threshold of a single molecule from an integer to a noninteger value has little effect on the steady state levels of AP-1 (results not shown). These changes can effect the activity profile of the proteins over time - allowing sustained (threshold = 0.0) or transient activity (0.0 < threshold < 1.0) after activation, or recapitulating protein lability and potential loss of signal. For example,in the case of EGF stimulation, the receptor has negative feedback encoded in the model. Therefore, increasing the thresholds of proteins downstream of EGFR to noninteger values affects the overall activity profiles of the molecules across time (results not shown). Increasing thresholds by integer values can have the same effect as node deletion when the number of activating (input) molecules is equal to the threshold. In general, increasing the threshold increases the required input value for signal propagation.

There are some important limitations in our approach. First, the model presented in this work did not consider any communication between the cells (autocrine or paracrine signals), or between the cells and the extracellular matrix. Contact inhibition and matrix attachment, and cell communication are important factors in cell cycle progression and we are considering their role in a cell-based model of liver tissues [7]. Second, we also recognize that the biochemical interaction network we used in this work is incomplete. Our primary goal was to develop a flexible modeling approach that can incrementally accommodate additional mechanistic information as it becomes available.

The BN used in this work was constructed semi-automatically from a molecular network topology, which was defined with little manual intervention using curated information on pathways. In future work we will evaluate the effects of additional pathways in the crosstalk network, for example, the interleukin protein family as well as the behavior of mito-inhibitors like Transforming Growth Factor Beta (TGF-β). For this work, we did not investigate alternative hypotheses on the signaling mechanisms of individual hepatocytes. The model development in [16] provides a method for investigating signaling differences between cell types. Understanding the signaling differences between cell types, such as, kinetic differences, is undoubtedly important in extrapolating cell line *in vitro *data to acute/chronic *in vivo *responses. Leveraging information from alternate pathway databases [49-51] should increase the descriptive power of our model, and is critical to decipher the role of molecular crosstalk in cellular responses to endogenous ligands and xenobiotics.

AP-1 is among several transcription factors important for cell cycle initiation/progression. In the current model we did not include gene expression regulated by the activation of transcription factors. Therefore, we chose AP-1 formation as the surrogate for downstream events, since it promotes cell cycle progression through increased expression of proteins, such as cyclin D1 [52], and antagonizes the function other molecules, such as p53 and p21 [53]. As we continue to develop this model, we can incorporate the activity of additional important transcription factors, such as Nuclear Factor kappa B (NF-κB) and Forkhead Box (FOX) proteins. Finally, most BN based approaches cannot directly relate simulation time steps to physiologic time intervals. This is an open problem in BN simulation and our approach is not immune to this issue, but we hope to address this in future work.

An advantage of asynchronous BN ensembles is that they can be simulated very rapidly. A single simulated treatment group (1000 cells/replicate) can be executed on a computer in minutes, while some of the more complex simulations, such as the reproducibility investigation (Figure 4) can be simulated on the scale of hours. However, since each cell is initialized/simulated to steady state individually, the approach is amenable to parallelization. We believe this efficiency will allow us to simultaneously investigate the role of molecular network topology using background knowledge on quantitative tissue level responses from experimental data.

## Conclusions

Systems biology approaches are vital for efficiently analyzing the effects of environmental contaminants on living tissues in order to evaluate the potential risk to public health. We developed asynchronous threshold Boolean network ensembles for translating mechanisms to *in vitro *and *in vivo *observations on molecular activity. The reproducibility of our modeling framework was evaluated by systematically analyzing the variability of our predictions across multiple simulations. We also showed that populations of hepatocytes can be simulated in this manner to predict experimentally observed quantitative responses. We believe that ensembles of Boolean networks can allow us to probe deeper mechanistic questions about the mode of action for chronic liver injury. We are testing this modeling approach as part of a broader computational and experimental effort aimed at estimating the putative effects of xenobiotics on the human liver by integrating chemical concentration, molecular pathways, cellular responses, and the role of cell-cell communication.

## Methods

### Threshold Boolean Network

We begin the definition of the cellular model with a biochemical interaction network as a signed, directed graph, *G(V,E)*, where *V *is the set of all vertices (or proteins/molecules) and *E *is the set of all edges (or reactions). Let *v *∈ *V*. Then, we define the set of all predecessors of *v*:(1)

For each edge *e_uv _*∈ *E *we have *Sign*(*e_uv_*) ∈ {+,-} where '+' indicates *u *is involved in the activation of *v *and '-' indicates *u *is involved in the inhibition of *v*. Now we define *A_v _*⊆ *P_v _*as the set of all activators of *v*. More formally,(2)

Likewise, we define *I_v _*⊆ *P_v _*as the set of all inhibitors of *v*,(3)

Furthermore, we let  and , and let *n *be the number of proteins in the graph. Additionally, we store the binary vector, , of the state (active or inactive) of every vertex at time *τ*. The state of a protein is dependent on the states of its predecessors. Therefore, we define a vector, , representing the threshold of activation for each vertex, a biologically inspired variable guiding the interplay predecessor vertices and protein activation. For the model, all thresholds were set to 0.0 with the exception of AP-1 formation, which is set to *θ*_*AP*-1 _∈ (1,2). This modification to the AP-1 threshold reflects the underlying biochemistry in that both c-Jun and c-Fos must be active for the activation of the AP-1.

Finally, we define the vector , which represents the probability of activation for each protein. For most models/proteins, there is a basal level of activity. We assume that individual BNs can have different protein activity profiles upon initialization (τ = 0), which allows for biological variability across the cell population.

Now, for any direct, signed graph, we formally define our model as follows:(4)

### Temporal Evolution of the Boolean Network

All other components of the model, *C^τ^*, are fixed during the simulation except for one, Δ^*τ*^, which is the state vector for ***v ***∈ *V *at a given time step. This value is determined by using the following threshold based logic:(5)

The steady state protein activity in a BN is expressed as the following state vector:(6)

### Model Calibration

In this model, the probabilities of activation for proteins, Φ, are considered a tunable parameter. The probabilities determine the state of each BN at τ = 0, which influences the dynamics of protein activation across the ensemble. We adjusted the values of Φ in three steps using experimental data where available. First, we assumed that in the absence of any treatment (i.e., experimental controls) the ligands, receptors, the adaptor proteins (GRB2 and SHC), the insulin receptor substrates (IRS and IRS-2), and non-ligand receptors (ErbB2 and ErbB3) have *φ *= 0.0. Second, the values of Φfor the remaining proteins in the network were increased until the predicted activity of AP-1 was close to the experimental level of BrdU incorporation in the control group (~1.5% DNA Synthesis [41]). For these proteins, the probability of activation *φ *= 0.0025 gave 1.49% ± 0.05 of AP-1 formation. Third, we assumed that the probability of activation of ligands in the model was related to the experimental concentration of the ligand. For EGF, we used the Hill function (Equation 8) to describe the relationship between probability of activation, *φ_EGF_*, and treatment concentration (in *ng/ml*).(8)

We used the BrdU concentration response data [41] to estimate the parameters for Equation 8. In order to use the Nelder-Mead algorithm to make a maximum likelihood estimation of *V*_max_, *K_A _*and *n*, we assumed that EGF activity corresponds to AP-1 activation. Although the network was encoded with negative feedback for the EGF receptor, representing the internalization and ubiquitination of this receptor, we make this assumption based on the simulation results with *φ_EGF _*= 1.0 (Figure 5a). The maximum likelihood estimates we found are *n *= 0.7, *K_A _*= 5.9*ng/ml *and *V*_max _= .28 (probability of activation).

### Simulating Populations of Cells

The ensemble of asynchronous threshold Boolean networks at a time step is represented as:

The aggregate activity of each protein across the ensemble at one time step, denoted as *v^τ^*, is calculated across *C^τ^*, as follows:(9)

Similarly the steady state activity of a protein across the ensemble is denoted as *v^T^*.

Hence, the coefficient of variation of the steady state protein activity is calculated as follows:(10)

### Implementation and Input Formats

The simulator is implemented in Python and the network model was produced interactively using Cytoscape. The molecular interaction network topology is defined in the Cytoscape exported format and protein information as well as quantitative parameters can be defined in the node attributes file (e.g., protein name, probabilities of activation, and threshold). The Python code as well as the network model are included (see Additional file 2 and Additional file 3).

## Competing interests

The authors declare that they have no competing interests.

## Authors' contributions

JJ designed and implemented the methods, performed the analysis, and wrote the manuscript; JW participated in developing the methods and writing the manuscript; IS supervised the development of the methods, implementation, analysis, and writing the manuscript.

## Supplementary Material

Additional file 1 **Evaluation of Network Behavior for Protein Knockouts**. The heatmap shows the simulation results for deleting individual proteins from the network. Each cell in the heatmap represents the mean protein activity at steady state relative to control across 20 replicates with 100 cells per replicate. The color intensity indicates the protein (x-axis) behavior at steady state relative to the baseline simulation (no protein knockout). The y-axis indicates the protein deletion. Click here for file

Additional file 2 **network_model.zip**. The Cytoscape export files for the crosstalk network model on AP-1 formation. These files are to be used in conjunction with the python_simulation_code. Click here for file

Additional file 3 **python_simulation_code.zip**. The source code to load the network model and produce the simulation results reported in the paper. Refer to the README file for instruction. Click here for file
