# Supplementary figures and images for: Simulating Quantitative Cellular Responses Using Asynchronous Threshold Boolean Network Ensembles
Source: BMC Syst Biol. 2011 Jul 11;5:109. doi: 10.1186/1752-0509-5-109 (PMC3224452; doi:10.1186/1752-0509-5-109)

**
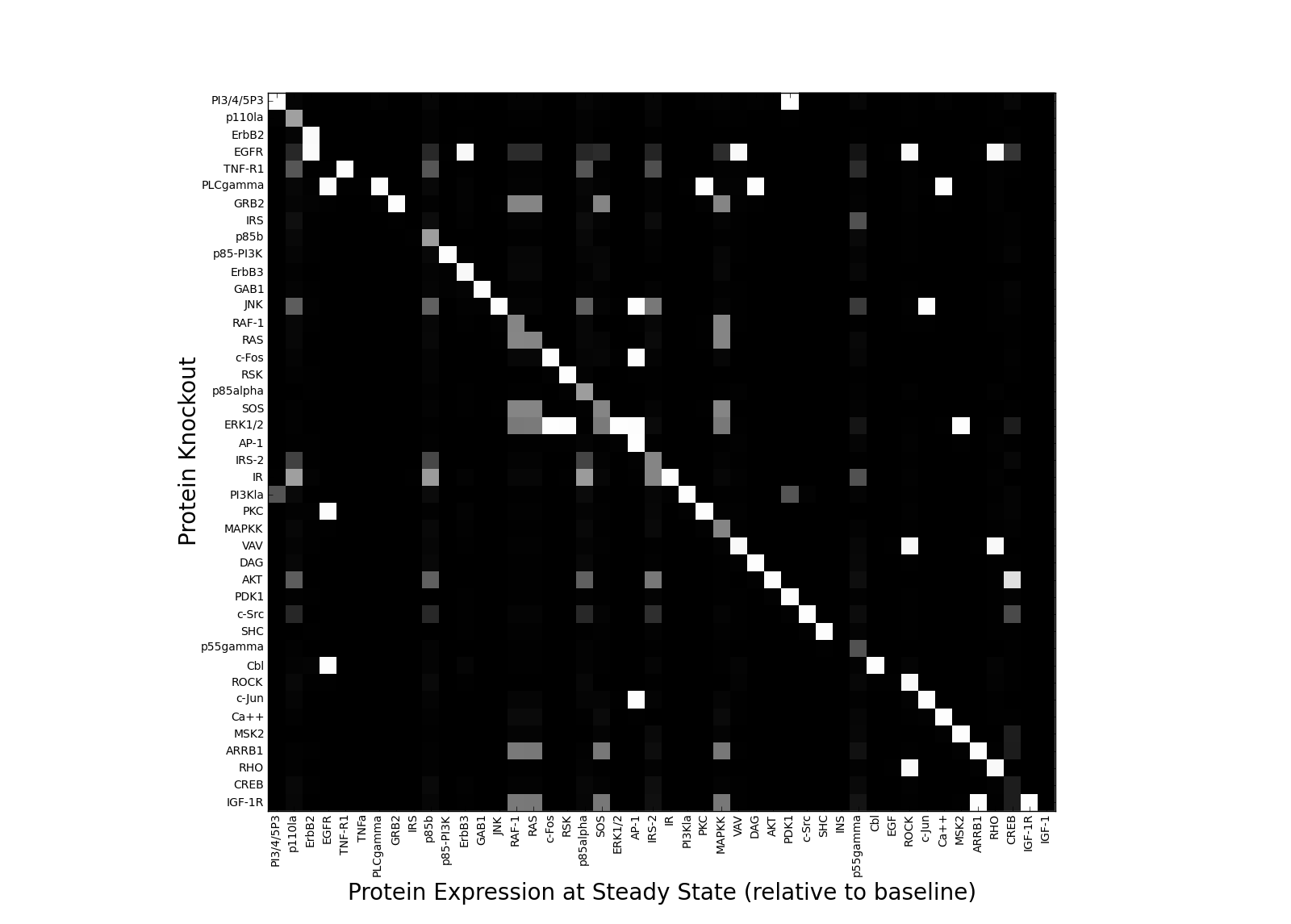
**

Supplement: Additional file 1 — Evaluation of Network Behavior for Protein Knockouts. The heatmap shows the simulation results for deleting individual proteins from the network. Each cell in the heatmap represents the mean protein activity at steady state relative to control across 20 replicates with 100 cells per replicate. The color intensity indicates the protein (x-axis) behavior at steady state relative to the baseline simulation (no protein knockout). The y-axis indicates the protein deletion. [file 1752-0509-5-109-S1.DOC]
